# Ecosystem size matters: the dimensionality of intralacustrine diversification in Icelandic stickleback is predicted by lake size

**DOI:** 10.1002/ece3.2239

**Published:** 2016-06-29

**Authors:** Kay Lucek, Bjarni K. Kristjánsson, Skúli Skúlason, Ole Seehausen

**Affiliations:** ^1^ Aquatic Ecology and EvolutionInstitute of Ecology and EvolutionUniversity of BernBaltzerstrasse 6CH‐3012BernSwitzerland; ^2^ Department of Fish Ecology and EvolutionEAWAG Swiss Federal Institute of Aquatic Science and TechnologyCenter for Ecology, Evolution and BiogeochemistryCH‐6047KastanienbaumSwitzerland; ^3^ Department of Animal and Plant SciencesUniversity of SheffieldSheffieldUK; ^4^ Department of Aquaculture and Fish BiologyHólar University College550SaudárkrókurIceland

**Keywords:** Ecological speciation, *Gasterosteus aculeatus*, multivariate evolution, speciation continuum

## Abstract

Cases of evolutionary diversification can be characterized along a continuum from weak to strong genetic and phenotypic differentiation. Several factors may facilitate or constrain the differentiation process. Comparative analyses of replicates of the same taxon at different stages of differentiation can be useful to identify these factors. We estimated the number of distinct phenotypic groups in three‐spine stickleback populations from nine lakes in Iceland and in one marine population. Using the inferred number of phenotypic groups in each lake, genetic divergence from the marine population, and physical lake and landscape variables, we tested whether ecosystem size, approximated by lake size and depth, or isolation from the ancestral marine gene pool predicts the occurrence and the extent of phenotypic and genetic diversification within lakes. We find intralacustrine phenotypic diversification to be the rule rather than the exception, occurring in all but the youngest lake population and being manifest in ecologically important phenotypic traits. Neutral genetic data further indicate nonrandom mating in four of nine studied lakes, and restricted gene flow between sympatric phenotypic groups in two. Although neither the phenotypic variation nor the number of intralacustrine phenotypic groups was associated with any of our environmental variables, the number of phenotypic traits that were differentiated was significantly positively related to lake size, and evidence for restricted gene flow between sympatric phenotypic groups was only found in the largest lakes where trait specific phenotypic differentiation was highest.

## Introduction

Instances of ecologically driven divergence between populations that may ultimately lead to speciation can be characterized along an evolutionary continuum from intraspecific variation with a single phenotypic and genotypic mode to bimodal distributions of phenotypic or genetic clusters with varying levels of reproductive isolation, and eventually phenotypically discrete and reproductively isolated species (Seehausen et al. 2008a; Hendry 2009; Nosil et al. 2009; Seehausen 2009; Nosil 2012). At the very early stage of the diversification process, phenotypic variation may be unimodally distributed (Doebeli and Dieckmann 2000; Hendry et al. 2009; but see Smith and Skúlason 1996; Smith et al. 1997). Divergent selection favoring phenotypes in the tails of the distribution can subsequently lead to the emergence of phenotypically differentiated groups, and a multimodal distribution of phenotypic variation can arise (Wright 1932; Gavrilets 2004; Leimar et al. 2008). This initial diversification may be promoted by phenotypic plasticity (West‐Eberhard 2003; Snorrason and Skúlason 2004; Pfennig and McGee 2010) and/or adaptive standing genetic variation (Barrett & Schluter, 2008; Lucek et al. 2014a; Marques et al. 2016). The release from interspecific competition and the associated relative increase of intraspecific competition may further promote evolutionary response to divergent selection (Yoder et al. 2010), which is especially expected in isolated and species‐poor locations where early colonists do not experience much competition from other species. Isolation may, however, also decrease the rate of diversification through its negative effects on the amount of standing genetic variation, where gene flow from outside may deliver important genetic variation but may also impede the emergence of reproductively isolated groups (Wright 1945; Seehausen et al. 2008b; Abbott et al. 2013). Overall, demographic, genetic, and environmental variation may all contribute to variation in the rate and extent of population divergence, and thus, progression along this evolutionary continuum can be arrested and even reversed at almost any time (Nosil et al. 2009).

The extent to which a species may diversify into several is thought to sometimes be limited by the number of available niches (Simpson 1953; Schluter 2000; Gavrilets and Vose 2005; Wagner et al. 2014). While the relationship between isolation, habitat heterogeneity, and the degree of diversification has been especially studied at large spatial and macroevolutionary scales (MacArthur and Wilson 1967; Ricklefs and Lovette 1999; Losos et al. 2009), it has less been investigated at the early stages of ecological divergence, that is, during the emergence of intraspecific differentiation (but see Woods et al. 2012 for an example). Here, we study such a case by comparing the degree of genetic and phenotypic diversification among several evolutionary relatively young three‐spine stickleback (*Gasterosteus aculeatus* species complex) lake populations with geographic isolation and physical properties of the respective lakes.

Three‐spine stickleback repeatedly colonized and adapted to freshwater habitats after the last glacial retreat (Hendry et al. 2013). In many cases, freshwater stickleback diverged ecologically from their ancestral marine species to different degrees, forming phenotypically distinct freshwater populations and species (McKinnon and Rundle 2002; Snowberg and Bolnick 2008; Hendry et al. 2009). There are several instances of freshwater stickleback diversification into distinct lake and stream ecotypes (Reimchen et al. 1985; Kaeuffer et al. 2012; Lucek et al. 2013; Ravinet et al. 2013a,b). Far less common are cases of intralacustrine diversification into multiple differentiated morphs or species (Hendry et al. 2013). This process has been especially investigated in Canadian coastal lakes some of which contain two distinct species, feeding predominantly on benthic or on limnetic food, respectively (Schluter and McPhail 1992; Rundle et al. 2000). The only other known cases of intralacustrine diversification are described from Alaska (Cresko and Baker 1996) and Iceland (Kristjánsson et al. 2002a; Ólafsdóttir et al. 2006). In the latter case, intralacustrine divergence into substrate‐associated morphs occurs (Jonsson 2002; Kristjánsson et al. 2002a,b). Icelandic lakes were likely colonized from similar marine populations sometime after the last glaciation, but they vary in their extent of geographic isolation from the sea as well as in surface area and depth. Thus, Icelandic lake stickleback allow to study the influence of the key island biogeography parameters, area and isolation, on the early stage of the diversification process.

Intralacustrine diversification among Icelandic stickleback has been found on six occasions (Jonsson 2002; Kristjánsson et al. 2002a; Ólafsdóttir et al. 2007b), with many studied focused on lakes Mývatn and Thingvallavatn (Kristjánsson et al. 2002a; Ólafsdóttir et al. 2007a; Ólafsdóttir and Snorrason 2009; Millet et al. 2013). In both lakes, stickleback formed phenotypically distinct substrate‐associated morphs: a lava morph and a mud morph that both occupy shallow water. In addition to these, a deep‐water dwelling morph that forages in *Nitella* algae meadows growing on mud substrate at water depths between 10 and 20 m depth occurs in Thingvallavatn (Sandlund et al. 1992a; Ólafsdóttir et al. 2007a). The morphs are distinct in terms of antipredator defense traits as well as in their feeding habits (Kristjánsson et al. 2002a; Doucette et al. 2004), and positive assortative mating between the Nitella and lava morphs has been observed in laboratory experiments (Ólafsdóttir et al. 2006). The morphs of Thingvallavatn have evolved since the retreat of the ice sheets ~8000 years ago (Sandlund et al. 1992b). Some other lakes though are much younger: Mývatn and its stickleback population are maximal, ~2300 years old (Einarsson et al. 2004), and other lakes are again much younger, such as man‐made Hraunsfjördur, a former lagoon that became landlocked in 1987 (Kristjánsson et al. 2002b).

Predicting that freshwater stickleback should become more genetically and phenotypically distinct with increasing geographic isolation from the ancestral marine population (MacArthur and Wilson 1967; Deagle et al. 2013), we first assessed to which degree Icelandic lake stickleback have phenotypically and genetically diverged from their ancestral marine population. We then tested whether environmental factors may explain variation in divergence. Secondly, we tested for the potential of intraspecific diversification into distinct phenotypic groups within each lake. Following the theory of island biogeography (MacArthur and Wilson 1967), and recent evolutionary extensions (Losos and Schluter 2000; Parent and Crespi 2006; Wagner et al. 2014), we predicted a larger number of differentiated phenotypes, increased phenotypic differentiation, including a larger number of trait dimensions in larger and more heterogeneous lakes, where potentially larger populations and more distinct ecological niches are available (Nosil & Sandoval, 2008; Gavrilets and Losos 2009; MacPherson et al. 2015).

## Material and Methods

### Sampling and data collection

**Figure 1:**
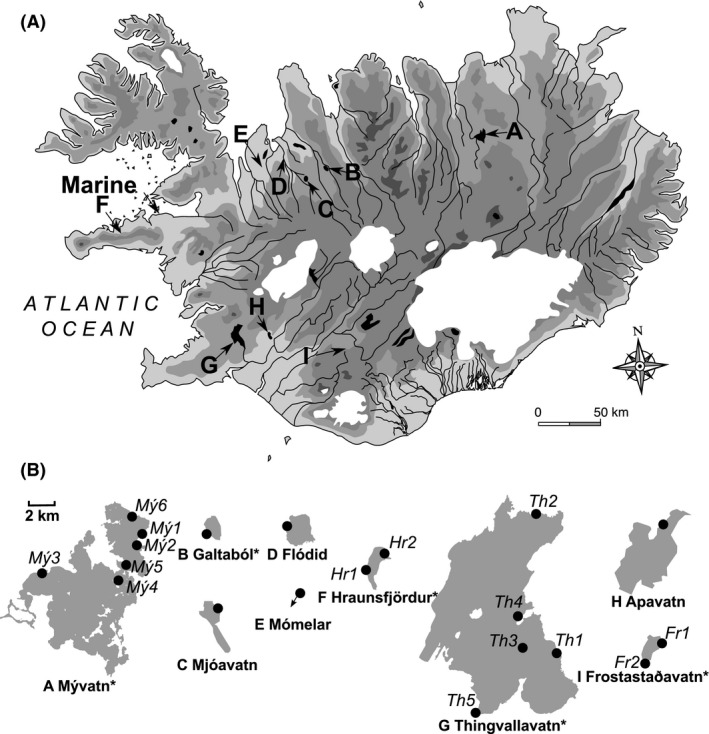
(A) Map of Iceland with sampled lakes indicated (modified from wikimedia© 2011). (B) Outline of sampled lakes drawn to the same scale (modified from openstreetmap project© 2011). Black dots indicate the site where sticklebacks were sampled, and asterisks indicate lakes for which substrate specific morphs have previously been reported (see main text and Table 1 for details).

In order to assess the effects of isolation and other environmental factors on the potential for within‐lake stickleback diversification, nine Icelandic lakes were selected that cover a wide range of environmental gradients, most notably distance from the sea, elevation above sea, and surface area (Fig. 1; Table 1). Substrate‐associated morphs have been described in five of these lakes: Thingvallavatn with three (Kristjánsson et al. 2002a; Ólafsdóttir et al. 2007a), and Mývatn, Galtaból, Frostastaðavatn, and Hraunsfjördur with two morphs each (Jonsson 2002; Kristjánsson et al. 2002a). In addition, a marine population from West Iceland (Breiðafjörður) was sampled representing a presumed ancestral population of Icelandic freshwater stickleback.

**Table 1:** Summary table for all sampling sites: distance from the sea, surface area, maximal lake depth and altitude (meters above sea level), lake position with its associated substrate, and sample size for each sex (*N*)

Lake		Distance from the sea [km]	Surface [km^2^]	Max depth [m]	Elevation (masl)	Latitude (N)	Longitude (W)	Substrate	*N*_females_	*N*_males_
Apavatn		71	13	2.5	64	64°11.70′	20°37.98′	Lava/vegetation	30	11
Flódid		15	2.1	1.5	11	65°29.45′	20°21.59′	Mud/vegetation	26	4
Frostastaðavatn	Fr1	137	2.3	11	581	64°01.42′	19°02.50′	Mud	21	40
	Fr2							Lava/vegetation	52	24
Galtaból		62	1.7	10	457	65°15.74′	19°44.47′	Lava/sand	29	28
Hraunsfjördur	Hr1	5	2.3	84	3	64°55.90′	23°01.66′	Mud	20	20
	Hr2							Lava	27	12
Mjóavatn		59	3.3	1.1	453	65°15.66′	19°48.04′	Lava/mud	32	29
Mómelar		64	0.03	2	142	65°25.29′	20°39.93′	Lava/mud	25	9
Mývatn	Mý1	23	37	4.5	286	65°37.99′	16°37.40′	Mud	14	16
	Mý2							Mud	8	22
	Mý3							Lava	13	17
	Mý4							Mud/vegetation	25	13
	Mý5							Mud/vegetation	22	18
	Mý6							Lava/mud	18	12
Thingvallavatn	Th1	57	82	114	107	64°09.01′	21°02.75′	Lava	15	7
	Th2							Lava	10	20
	Th3							Vegetation^1^	17	13
	Th4							Mud/vegetation	13	44
	Th5							Lava	8	9
Marine		–	–	–	–	65°02.66′	22°27.48′	Sand	31	14

^1^Sampled offshore between 10 and 20 m depth in *Nitella* sp. meadows.

Individuals were sampled from 21 locations among the nine lakes between August and September 2010 (Table 1) using minnow traps and by hand netting. All fish were euthanized with an overdose of clove oil and stored in ethanol. In addition, a fin clip was taken for genetic analyses. The number of sampling locations within a single lake ranged from 1 to 6, where cases with different locations within the same lake reflect previously established sampling locations that differ in their substrates types (Kristjánsson et al. 2002a, 2004). For lakes with a single sampling location, traps were placed such as to sample as much of the available substrate variation as possible, but individuals from all traps were subsequently pooled. Diversity of substrate types was qualitatively recorded. Sample size per site ranged from 17 to 71 individuals (mean: 40 ± 15 SD) with a total of 845 individuals.

### Genetic analysis

DNA for all individuals was extracted using a 10% Chelex solution, following the manufacturers protocol (Bio‐Rad, Cressier, CA, USA). Nine microsatellite markers (Gaest66, Stn26, Stn30, Stn96, Stn130, Stn173, Stn174, Stn185, and Stn196), each from a different chromosome, were amplified in one multiplex kit (Lucek et al. 2014a). Three of these markers (Stn26, Stn96, and Stn130) are associated with QTL for spine lengths in North American stickleback (Peichel et al. 2001), and Stn130 was additionally found to be associated with the number of gill raker in Belgian stickleback (Raeymaekers et al., 2007). Consequently, these markers are predicted to lead to genetic substructure if they are linked to a phenotype under divergent selection in contrast to neutral markers. Alleles were visualized on an ABI 3130XL and scored with genemapper 4.0 ( Applied Biosystems, Zug, Switzerland). Sex of each individual was determined using a molecular marker (*Idh*) yielding either one or two bands (separated by 30 bp) in females and males, respectively, following Peichel et al. (2004).

In total, 791 of 845 individuals measured for morphological traits were successfully genotyped. Molecular sexing failed for seven individuals, which were omitted from all analyses that required information on sex. Linkage disequilibrium among all marker pairs were calculated for each lake and the marine population separately using genepop 4.2 (Rousset 2008), followed by a sequential Bonferroni correction. Heterozygosity, pairwise *F*_ST_ between each lake (pooling all sample sites within a lake) and the marine population, the pairwise *F*_ST_ among all sites within each lake, and the *F*_ST_ between identified phenotypic groups within each lake were calculated using genodive 2.0 (Meirmans and Van Tienderen 2004). Significance levels were estimated for all *F*_ST_ using 1000 bootstrapped replicates as implemented in genodive. *F*_ST_ calculations were performed using either all loci combined or separately for putatively neutral or putatively QTL‐linked markers. To then test whether pairwise *F*_ST_ values based on neutral marker would differ from QTL‐linked marker, the respective *F*_ST_ values were compared with paired *t‐*tests. Because pairwise *F*_ST_ did not differ between the two types of markers (pairwise *F*_ST_ between the marine population and each lake: paired *t*_1,8_ = 0.03, *P *=* *0.975; pairwise *F*_ST_ between identified phenotypic groups within lakes: paired *t*_1,11_ = 0.04, *P *=* *0.972), all markers were pooled for all further genetic analyses. Linear models were then used to test for a linear relationship of heterozygosity or the pairwise *F*_ST_ between each lake and the marine population with environmental variables (elevation and distance from the sea; Table 1). In addition, the overall level of inbreeding (*F*_IS_) was estimated for each lake and for the marine population using an AMOVA approach with 10,000 bootstrapping replicates to infer potential genetic substructure. Global *F*_IS_ was also calculated for each identified phenotypic group of each sex. The genetic structure within each lake was estimated using an admixture model implemented in structure 2.3.3 (Falush et al. 2007) with 30,000 burn‐in steps followed by 300,000 MCMC steps. The simulation was performed assuming 1–6 genetic clusters (K) with 10 replicates for each assumed K. The simulation was run for the marine population and also separately for each lake, either pooling both sexes or separately for each sex when more than 20 individuals were available. The optimal number of genetic clusters was determined by investigating the individual assignment plots, the log‐likelihood values of each run and their variation among runs for the same K. To establish the genetic relationship among the populations sampled from different sites and lakes, a tree was calculated based on Cavalli‐Sforza distances of allelic frequencies using a neighbor‐joining algorithm implemented in the program PHYLIP 3.69 (Felsenstein 2012). Significance levels of the tree topology were estimated using 1000 bootstrapped resampling replicates.

### Phenotypic measurements

Sixteen ecologically relevant linear morphological traits were measured to the nearest 0.01 mm using a digital caliper (see Reimchen et al. 1985; Schluter and McPhail 1992; Kristjánsson et al. 2002a; Mori and Takamura 2004; Berner et al. 2008 and references therein). These traits were related to either antipredator defense (lengths of the first and second dorsal spine; lengths of the pelvic spine and the pelvic girdle), feeding ecology (head length; upper jaw length; snout length and width; eye diameter), or general body shape (standard length; width of the pelvic girdle; body depths measured after the first and second dorsal spine; caudal peduncle length; basal lengths of the anal, dorsal, and pelvic fin). Two additional traits related to feeding ecology were measured (Berner et al. 2008): The length of the second gill raker, as counted from the joint of the dorsal arch bone of the first gill arch, and the length of the lower gill arch were measured using a micrometer mounted on a dissection microscope. Because all traits were significantly related to standard length (results not shown), a size correction was applied using the residuals of a regression of each trait against SL for each lake separately to remove effects of potential differences in allometry between lakes. Lastly, both sagittal otoliths, calcium carbonate structures in the inner ear that show seasonal rings, were extracted from each individual. Winter rings were counted at 40x magnification using a microscope to estimate the age of each individual (Zeller et al. 2012).

### Estimating phenotypic changes along the marine–freshwater transition

Phenotypic diversity in each lake was estimated as the amount of morphospace occupied, defined as the size of the 95% confidence ellipsoid for all individuals of a particular lake on the two main principal component (PC) axes, using all size‐corrected linear traits together. Relative ellipse size was calculated using a custom made script based on an implementation in the car package (Fox and Weisberg 2011) in R 2.15.1 (R Core Team 2012). Subsequently, the morphospace estimates for lake populations were scaled by the highest observed value. To overcome potential artifacts due to different sample sizes (Table 1), the analysis was repeated using a resampling approach with 1000 replicates, where for each replicate 25 individuals were randomly selected without replacement for each lake and marine population. The scaled estimates of morphospace were then regressed against sample size, lake characteristics (distance from the sea, surface area, elevation), and against the observed heterozygosity using linear models.

To estimate the overall degree of phenotypic differentiation between the ancestral marine population and each lake, pairwise *P*_ST_, unitless proportional measures of pairwise phenotypic differentiation that are analogous to the measure of pairwise genetic differentiation (*F*_ST_) were calculated. *P*_ST_ was based on the scores of the first PC axis of each lake population and the marine population. Calculations followed Kaeuffer et al. (2012), where *P*_ST_ values and their 95% confidence intervals were estimated using a resampling approach with 1000 replicates. Obtained *P*_ST_ values were then regressed against distance of lake from the sea, lake elevation as well as the pairwise *F*_ST_ against the marine population.

### A cluster‐based approach to estimate intralacustrine diversification

To assess the number of distinct phenotypic groups within each lake as well as within the marine population, a cluster‐based analysis was employed. The best clustering method for the morphological data was first determined using clvalid (Brock et al. 2008) and rankaggreg (Pihur et al. 2009) for all lakes and the marine population. clvalid and rankaggreg identified the UPGMA algorithm based on Euclidean distances as the best fitting algorithm for seven of ten cases and it was within the top three among the others. Therefore, UPGMA was used for all subsequent cluster analyses.

The number of statistically supported multivariate phenotypic groups was then determined using a dynamic hybrid tree cut (Langfelder et al. 2008). In short, this method is based on a bottom‐up algorithm which first identifies preliminary clusters depending on a given minimal cluster size, the distance and distinctiveness of its neighboring objects, and the connectivity of branches within a cluster. In a second step, previously unassigned objects are tested for their proximity to the preliminary clusters and get either assigned or not (see Langfelder et al. 2008 for details). Because this method is based on tree topology without prior assumptions on the number of inferred clusters, it provides an unbiased estimate for the number of clusters that are present in a given data set. As stickleback can be sexually dimorphic and form distinct ecotypes at the same time (Cooper et al. 2011), the cluster‐based analyses were performed separately for each sex per lake. For all lakes, the settings were as follows: minimal cluster size: eight individuals; maximal scatter: 0.75; minimal gap size: 0.25; and maximal distance for assignment: 0.90. The last three values relate to the fraction between the maximal node height observed in the UPGMA tree and the 5th percentile of all node merging heights. The obtained clusters were stable unless extreme values were taken (results not shown). A minimal cluster size of eight was chosen to allow for subsequent statistical analyses on the identified groups. This approach gives a conservative estimate of the minimum number of groups, as clusters with only few individuals are omitted from subsequent analyses.

Identified intralacustrine groups were subsequently tested for an association with age, based on otolith readings and size (standard length) using an ANOVA. In addition, chi‐square tests were employed to test whether individuals assigned to distinct groups were not randomly distributed among sampling sites and substrate types in lakes where such information was available. Statistical phenotypic differentiation among groups was furthermore tested using a MANOVA, including all measured phenotypic traits and using *group* as a factor. Individual trait differentiation between groups and hence the dimensionality of differentiation were further investigated with an ANOVA for each trait, using a Benjamini and Yekutieli correction to account for multiple testing (Narum 2006). The trait dimensionality was subsequently tested for an association with lake area, elevation, maximal lake depth, and distance from the sea using a linear model with sex as a fixed factor. In cases where multiple phenotypic groups were identified for both males and females, Mahalanobis distances were calculated among all groups and visualized in a dendrogram to further infer whether the distinct phenotypic groups cluster by sex or morph. Lastly, to visualize the multivariate distribution of individuals according to the identified cluster, a PC analysis was performed using all linear traits separately for each lake and sex.

## Results

### The marine–freshwater transition

**Figure 2:**
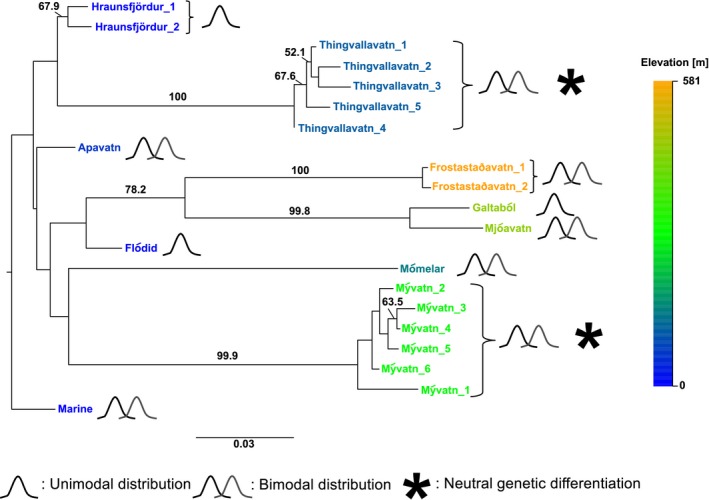
Genetic relationships among Icelandic populations of lake stickleback with a marine population as outgroup. Neighbor‐joining tree using Cavalli‐Sforza distances among sampling sites included in this study (see Table 1), calculated from allele frequencies at nine microsatellite loci. Numbers beside nodes indicate percent bootstrap support based on 1000 resampling replicates. Bootstrap values below 50% are not shown. Note that the deep part of this tree is effectively an unresolved polytomy, consistent with independent colonization from the sea for every lake except the three high‐altitude lakes Frostastaðavatn, Galtaból, and Mjóavatn, suggestive of an earlier colonization event of these lakes during the early phase of the isostatic adjustment of Iceland during the melting of the Icelandic ice sheets (Le Breton et al. 2010). Symbols depicting bimodal distributions indicate cases, where two phenotypic clusters were found that differ statistically from each other (see Fig. 4). Asterisks indicate cases where neutral genetic differentiation was found between modes based on pairwise *F*_ST_ and STRUCTURE (see Table 1).

The pairwise *F*_ST_ of lake populations against the marine population showed a significant positive relationship with elevation of lakes above sea level (*R* ^2^ = 0.868, *F*_1,7_ = 45.5, *P *<* *0.001), whereas the relationship of the observed heterozygosity within a lake with elevation was negative (*R* ^2^ = 0.787, *F*_1,7_ = 25.9, *P *=* *0.001). Neither of these was significantly related to the distance of a lake from the sea, but elevation and distance from the sea were positively related to each other (*R* ^2^ = 0.456, *F*_1,7_ = 5.9, *P *=* *0.046). The pairwise *F*_ST_ among the three lakes at relatively low elevation (i.e., <100 m above sea level; Table 1) and between any of these and the marine population was low (*F*_ST_ < 0.05, Table S1), whereas higher elevation lakes are all more strongly differentiated from the sea (*F*_ST_ > 0.15) and pairwise *F*_ST_ among these is high (*F*_ST_ > 0.30), with the exception of Galtaból and Mjóavatn (*F*_ST_ = 0.146; Table S1). In the population tree, all geographically separated high‐elevation lakes reside on long branches, whereas the three low‐elevation lakes (Apavatn, Flódid, Hraunsfjördur) and the marine population all sit on short branches (Fig. 2).

### Evidence for an increase in phenotypic variation after colonization of lakes

**Figure 3:**
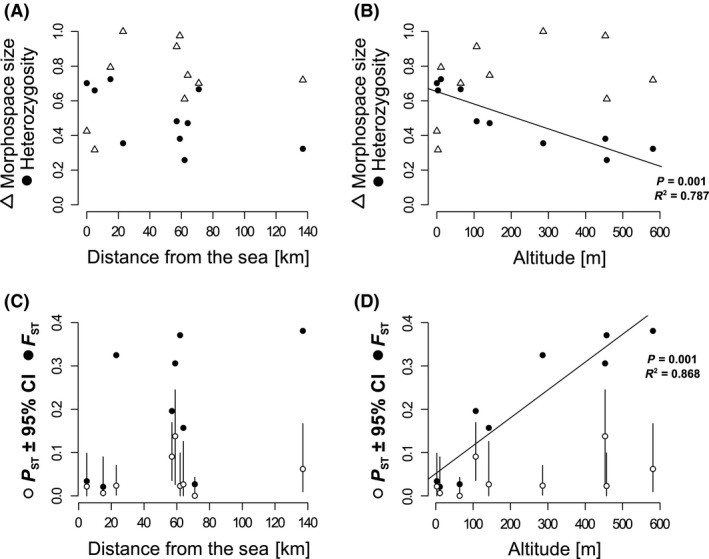
Genetic and phenotypic variability, as well as genetic and phenotypic differentiation from the ancestral marine population, plotted against the distance from the sea and against elevation (meters above sea level): (A) observed heterozygosity (black dots) and the relative size of occupied morphospace (triangles; see text for details) against the distance from the sea; (B) observed heterozygosity (black dots) and the relative size of occupied morphospace (triangles) against elevation; (C) pairwise *F*_ST_ (black dots) and *P*_ST__ _± its 95% confidence interval – CI (white dots) – of each lake against the marine population against the distance from the sea; (D) pairwise *F*_ST_ and *P*_ST_ ± its 95% confidence interval – CI – plotted against elevation. Regression coefficients and their significances are indicated for significant linear models.

The relative morphospace occupied by stickleback within each lake did not differ whether only females, males, or both sexes combined were analyzed, or whether the marine population was included or excluded from the PCA (results not shown). Therefore, only the average scaled estimates for the morphospace volume are shown that are based on the two main PC axes (accounting for 31.6% and 17.1% of the total variation, respectively) of the PCA comprising all individuals (Fig. 3A and B). The morphospace occupied by stickleback in each lake and in the marine population was not related to sample size (*R* ^2* *^= 0.193, *F*_1,8_ = 1.9, *P *=* *0.204). The Mývatn population showed the largest phenotypic variation, whereas the marine population and Hraunsfjördur, a recent marine isolate, were the least variable, occupying 42.5% and 31.7% of the size of Mývatn, respectively, on PC1 and PC2.

The colonization of lakes from the sea was associated with an increase in phenotypic diversity as lake stickleback occupied a larger amount of the common morphospace than the marine population (one‐sample *t*‐test: *t*_1,8_ **= 4.70, *P *=* *0.002), which was also true when each population was resampled to 25 individuals each (*P *=* *0.030), when Hraunsfjördur was excluded (one‐sample *t*‐test: *t*_1,7_ **= 7.74, *P *<* *0.001) or treated as a marine population (one‐sided *t*‐test: *t*_1,8_ **= 5.96, *P *=* *0.004). The morphospace of lake populations was not significantly associated with the distance from the sea, elevation, lake surface area, the maximum lake depth or the observed heterozygosity, and none of these results changed when Hraunsfjördur was included or not (all *P *>* *0.10, results not shown).

Phenotypic differentiation from the marine population, based on *P*_ST_ was strongest in Mjóavatn (*P*_ST_ = 0.138, 95% CI: 0.026–0.244, *P *=* *0.012) and Thingvallavatn (*P*_ST_ = 0.091, 95% CI: 0.035–0.169, *P *=* *0.013). *P*_ST_ values were associated neither with the distance from the sea (*R* ^2* *^= 0.089, *F*_1,7_ = 0.7, *P *=* *0.435) nor with elevation (*R* ^2* *^= 0.232, *F*_1,7_ = 2.1, *P *=* *0.190; Fig. 3). *P*_ST_ and pairwise *F*_ST_ between each lake population and the marine population were not statistically related (*R* ^2* *^= 0.224, *F*_1,7_ = 2.0, *P *=* *0.198).

### Evidence for phenotypic differentiation within lakes

**Figure 4:**
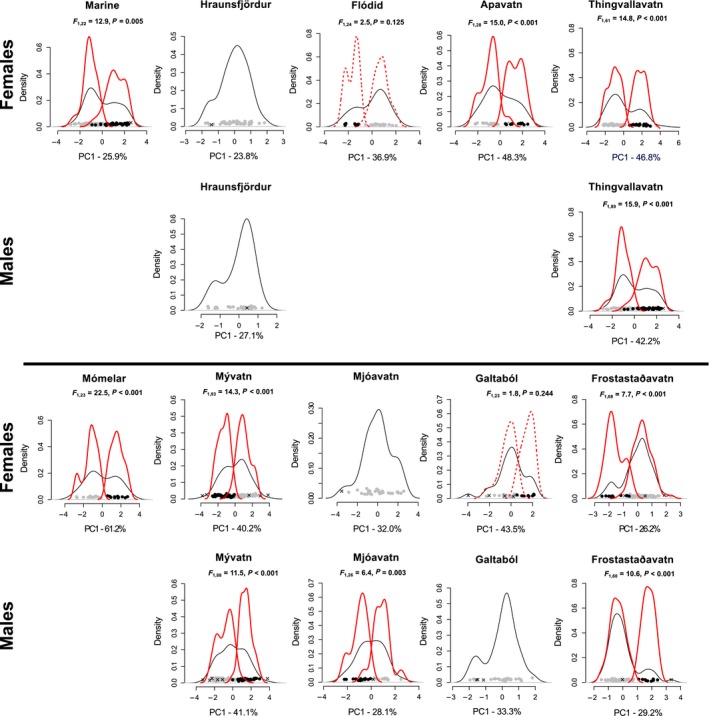
Kernel density function of the PC1 scores in each lake calculated for females and males separately. Kernel densities are shown for all individuals combined (black line) or separately for each identified multivariate mode (red line for cases where the MANOVA was significant, dashed line for nonsignificant cases). Crosses indicate individuals that were excluded by the clustering algorithm (see text for details). Above the density plots we indicate the *P* values between modes based on a MANOVA for all traits using clusters as factor. Empty panels indicate cases where sample size was too small to perform a clustering analysis. Note that the PC1 axis only reflects the major axis of multivariate trait variation and may thus slightly differ from the multivariate cluster analysis. Lakes are sorted by increasing elevation.

Combining the dynamic tree cut method and lake‐specific MANOVAs, distinct phenotypic groups and hence a bimodal phenotypic distribution were identified among females in five of nine lakes (Figs. 2, 4): Thingvallavatn, Mývatn, Mómelar, Frostastaðavatn and Apavatn; and also in the sea. In lakes Flódid and Galtaból, the dynamic tree cut found two groups, but the MANOVA did not support these differences (Fig. 4). No signal of bimodality among females was found in Hraunsfjördur or Mjóavatn. Due to the restricted sample sizes for males, we could apply the dynamic tree cut only to six lakes. No deviation from phenotypic unimodality was found among males in Galtaból and Hraunsfjördur. For males in lakes Frostastaðavatn, Mjóavatn, Mývatn, and Thingvallavatn, bimodal distributions were found and further confirmed by the MANOVA (Fig. 4). Hence, significantly differentiated phenotypic groups of stickleback were found in lakes Thingvallavatn, Mývatn, Frostastaðavatn, Mómelar, and Apavatn and in the sea, to some degree in Mjóavatn (only in males), but not in Hraunsfjördur, Flódid, or Galtaból. No indications for more than two phenotypic groups were found within either sex in any of the lakes. As indicated by the Mahalanobis distances among groups and sexes (Fig. S1), the two groups found in either sex separately in lakes Thingvallavatn, Mývatn, and Frostastaðavatn correspond to the same two morphs. No associations were found between the number of phenotypic groups in a lake (1 or 2) and any of the available environmental variables (all *P* > 0.300), which was also true when Hraunsfjördur was excluded (results not shown).

**Figure 5:**
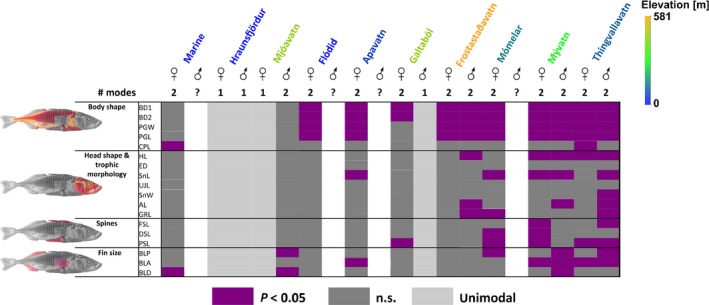
The ecological speciation continuum in Icelandic lake stickleback. Graphical representation of the phenotypic differentiation between the phenotypic clusters based on ANOVAs performed for each lake and sex (see main text for details and Table S3 for the actual statistical values). Purple color represents cases with significant differentiation (*P *<* *0.05), following a Benjamini and Yekutieli correction. Cases with a unimodal phenotypic distribution are highlighted in pale gray, whereas cases with low sample sizes are given in white. Abbreviations are as follows: BD1, body depth after the 1st dorsal spine; BD2, body depth after the 2nd dorsal spine; CPL, caudal peduncle length; PGW, pelvic girdle width; PGL, pelvic girdle length; HL, head length; ED, eye diameter; SnL, snout length; UJL, upper jaw length; SnW, snout width; AL, gill arch length; GRL, length of the second gill raker; FSL, length of the 1st dorsal spine; SSL, length of the 2nd dorsal spine; PSL, length of the pelvic spine; TLP, total length of the pelvic fin; BLA, basal length of the anal fin; BLD, basal length of the dorsal fin.

**Figure 6:**
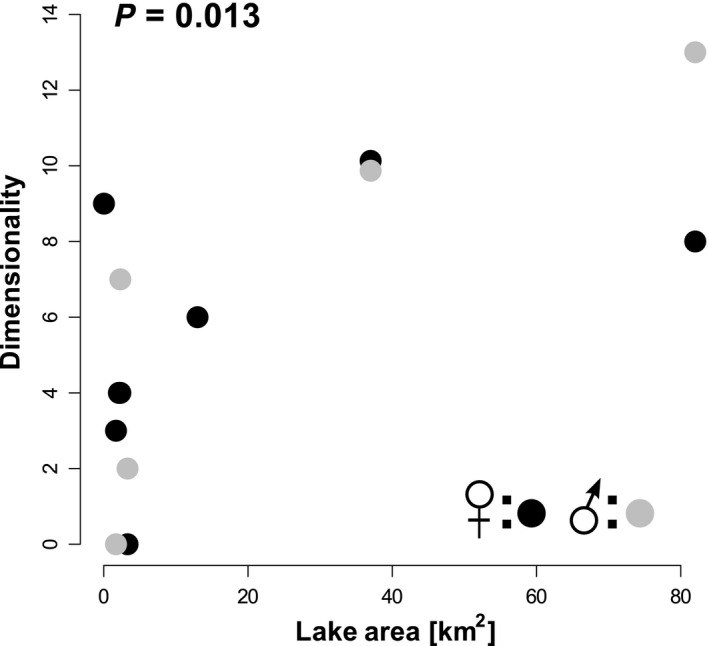
Relationship between lake area (km^2^) and the dimensionality of sympatric phenotypic differentiation. Dimensionality is measured as the number of significant differences between identified phenotypic clusters for both females (black) and males (gray; see Fig. 5 and Table S3). The *P* value derives from linear model using sex as a fixed factor (see main text for details).

The trait‐based ANOVAs indicate evidence for parallelism in trait divergence between sympatric morphs in different lakes (Fig. 5; Table S3). Differentiation occurs especially in body shape‐related traits (body depth and the pelvic girdle structure) wherever evidence for two groups within a lake was found with the same two traits being significantly differentiated between them (i.e., *P *<* *0.05; Fig. 5, Table S3). Although phenotypic divergence occurs also for head shape and defense‐related traits as well as fin sizes, the traits involved differ among lakes (Fig. 5). Interestingly, the traits that are divergent between the two female morphs in the marine population are mostly distinct from those that are divergent among sympatric lake morphs. The number of traits statistically differentiated among sympatric morphs after a Benjamini and Yekutieli correction, and hence, the dimensionality of phenotypic differentiation was greatest in the two largest lakes (Mývatn: females – 11; males – 10 of 18 traits; Thingvallavatn: females – 8; males – 14 of 18 traits). Similarly highly dimensional differentiation was found for females in Mómelar (9 of 18 traits), whereas fewer traits were significantly differentiated between groups in all other lakes (Fig. 5; Table S3). The number of statistically differentiated traits for all lakes that had two phenotypic morphs (Fig. 5; Table S3) was positively related to lake surface (*F*_1,10_ = 9.0, *P *=* *0.013; Fig. 6), but not with any other variable (i.e., elevation, maximal lake depth, distance from the sea; all *P *>* *0.050), based on linear models using sex as a fixed factor.

### Ecological associates of phenotypic differentiation

In a few lakes, the phenotypic groups that we identified were differentiated in standard length, age, or the substrate type from which they were sampled: For Frostastaðavatn, individuals assigned to the different female morphs differed in size (*F*_1,68_ = 4.8, *P *=* *0.032) and were nonrandomly distributed between mud and lava substrate ($\chi_{1,3}^{2}$ = 30.27, *P *<* *0.001). Individuals in the more abundant group derive mainly from the lava substrate and were smaller than individuals from the less abundant group where half of the individuals derived from the mud substrate. Individuals from the two male morphs were also nonrandomly distributed between substrate types ($\chi_{1,3}^{2}$ = 30.74, *P *<* *0.001). Males of the two morphs differed in age (*F*_1,60_ = 4.3, *P *=* *0.042), where the mean age of the less abundant group was 2.1 years as opposed to 1.8 years in the more abundant group. For both sexes, individuals in the more common group (lava substrate) had longer heads and slender bodies, where males had moreover elongated first dorsal spines and gill rakers.

In Mývatn, substrate (females: $\chi_{1,5}^{2}$ = 11.19, *P *=* *0.048; males: $\chi_{1,5}^{2}$ = 12.26, *P *=* *0.031) and sampling site (females: $\chi_{1,11}^{2}$ = 33.01, *P *<* *0.001; males: $\chi_{1,11}^{2}$ = 29.60, *P *=* *0.002) were both significantly different between the morphs. Interestingly, individuals from the genetically distinct site Mý1 (Table S2 and below) were phenotypically almost exclusively assigned to a single phenotypic cluster (100% for females and 77% for males), where individuals had shorter heads, snouts, fins, and spines and deeper bodies (Table S3). Lastly, age differed between the phenotypic groups that we found among females in the marine population (*F*_1,22_ = 6.3, *P *=* *0.020), all collected at the same site over the same substrate.

### Support for neutral genetic differentiation

Following a sequential Bonferroni correction, we found significant linkage disequilibrium only between the putatively neutral markers *Stn30* and *Stn173* in lakes Mývatn and Mómelar. structure found indication for genetic substructure in Mývatn (Table 2; Fig. S2), where some individuals showed more than 80% assignment probability to one or the other genetic cluster assuming *K* = 2 (Fig. S2). However, no individual was entirely assigned to either cluster. structure failed to detect additional genetic clusters when K was further increased. Using *K* = 2, the pairwise *F*_ST_ between the individuals that were assigned with ≥75% probability to the less abundant cluster (shown in red in Fig. S2) and all other individuals was higher (*F*_ST_ = 0.151, *P *<* *0.001) than any pairwise comparison between sites within Mývatn (range of *F*_ST_ 0–0.119; Table S2). Individuals with high assignment probability to the less abundant genetic cluster were mainly sampled from the two sites with muddy substrate (Mý1 and Mý2; Fig. 1), where the population at Mý1 seems almost entirely composed of the less common genetic cluster and Mý2 appears to have relatively even numbers of individuals belonging to both genetic clusters. Individuals from Mý1 are moreover genetically differentiated from other Mývatn sampling sites (Table S1), but no pattern of isolation by distance was found within Mývatn (Mantel test: *r *=* *−0.091, *P *=* *0.321).

**Table 2:** Summary table for population genetic indices calculated for each lake and the marine population. Indicated are the sample size (*N*) of genotyped individuals per sex and the total number of available individuals being genotyped for nine microsatellites (note: For some individuals, sex could not be determined). *K* indicates the number of genetic clusters identified by structure for cases where >20 individuals were available. Additionally, the global inbreeding coefficient (*F*_IS_) for all individuals within each studied system and separately for each sex, the global *F*_IS_ as well as the pairwise *F*_ST_ between the identified sex specific phenotypic modes within a lake are given (see main text for details)

Site	Sample size			structure			Global *F*_IS_			*F*_ST_ among modes	
	*N*_females_	*N*_males_	*N*_all_	*K*_females_	*K*_males_	*K*_all_	Females	Males	All	Females	Males
Apavatn	30	11	41	1	–	1	0.039	0.012	0.031	0.008	–
Flódid	26	4	30	1	–	1	0.014	0.000	0.008	0.001	–
Frostastaðavatn	73	58	131	1	1	1	−0.067	−0.064	−0.065	−0.015	0.018
Galtaból	27	26	53	1	1	1	−0.088	0.104†	0.009	−0.005	–
Hraunsfjördur	42	30	72	1	1	1	0.074**	0.013	0.048*	–	–
Mjóavatn	17	25	42	–	1	1	0.025	0.030	0.026	–	−0.009
Mómelar	25	9	34	1	–	1	0.011	0.132†	0.070*	−0.015	–
Mývatn	93	95	195	1	1 (2)^1^	1 (2)^1^	0.045*	0.103***	0.077***	0.002	0.006
Thingvallavatn	51	83	148	1	1	1	0.040†	0.035†	0.043**	0.000	0.009*
Marine	31	14	45	1	–	1	0.029	0.022	0.024	0.012	–

^1^Although no clear clustering was achieved, the structure runs indicate a potential substructure into two clusters (see Fig. S2 and main text for details).

***P <* *0.001; **P <* *0.01; *P <* *0.05; †0.05 < *P *<* *0.10.

No genetic structure was detected in any other lake using structure (Table 2). Among the sympatric morphs that were identified, only the two male groups from Thingvallavatn differed significantly (*F*_ST_ = 0.009, *P *=* *0.035; Table 2). However, global *F*_IS_ was significant in Mývatn, Thingvallavatn, Mómelar, and Hraunsfjördur (Table 2) using all microsatellite markers, indicating the potential for some nonrandom mating. Global *F*_IS_ was similarly significant in all but one of the aforementioned lakes (Mómelar) when putatively QTL‐linked markers were excluded. Lastly, the *F*_ST_ between Thingvallavatn sites Th3 and Th5 (*F*_ST_ = 0.026) and the two sampling sites in Hraunsfjördur (*F*_ST_ = 0.007) were significant using all genetic markers (Table S2). The latter is in concordance with a previous study on stickleback from Hraunsfjördur collected at the same locations, where stickleback differed phenotypically between sites (Kristjánsson et al. 2002b).

## Discussion

Several theoretical and empirical studies of sympatric diversification suggest an evolutionary continuum along which ecologically differentiated sympatric populations may fall, with a unimodal trait distribution at one end and the emergence of multimodality and eventually phenotypically and genetically distinct species at the opposite end (e.g., Doebeli and Dieckmann 2000; Hendry et al. 2009; Seehausen 2009; Feder et al. 2012; Nosil 2012; Seehausen et al. 2014). Although several environmental factors have been identified that underlie the extent of species diversification during adaptive radiations, that is, the proliferation of a single ancestral lineage into a variety of species adapted to different ecological niches (e.g., Losos and Schluter 2000; Parent and Crespi 2006; Wagner et al. 2014), less is known about the factors that determine the extent of differentiation during the early stages of ecological speciation (Nosil et al. 2009; Nosil 2012; Seehausen et al. 2014; but see Woods et al. 2012). Studying lake populations of Icelandic three‐spine stickleback, we found that the colonization of freshwater lakes from the sea was generally associated with an increase in phenotypic variation. We found phenotypic variation was unimodal in some lakes, but bimodal in other potentially older lake populations at higher elevation. The distinct phenotypic groups within a lake were in one case associated with occupation of different substrate types, in two cases with sampling site and in one case with body size and fish age. Although variation in lake size and lake depth did not significantly explain the observed phenotypic variation, the phenotypic dimensionality of differentiation between sympatric morphs was positively related to lake surface area (Fig. 6), our proxy for ecosystem size.

We found evidence for differentiation among morphs at neutral genetic markers only in the two largest lakes, which may signal an advanced state in the process of ecological speciation (Hendry 2009; Seehausen 2009; Feder et al. 2012; Nosil 2012). The degree of genetic differentiation (*F*_ST_) of lake populations from the marine population increased with elevation of lakes above sea level (Figs. 2, 3), which is consistent with a pattern of increasing isolation (Deagle et al. 2013). Three of our studied lakes are at distinctly higher elevation than the others, and these clustered together despite being geographically distant. This pattern may reflect different colonization waves by stickleback to Icelandic freshwater lakes, where depending on the distinct climatic history during the last glaciation, some upland lakes (>450 m) became available for colonization when the others may still have been under the sea (Le Breton et al. 2010). Also, the level of heterozygosity and thus standing genetic variation within lakes decrease with elevation, which likely reflects drift and genetic bottlenecks. Moreover, if neutral marker diversity reflects diversity at ecologically relevant loci, then this may predict that the potential for adaptive diversification for Icelandic lake stickleback is highest at intermediate elevations, where current gene flow from the sea is absent or weak, but standing genetic variation is moderately high (Kawecki and Ebert 2004; Räsänen and Hendry 2008). Consistent with this, we find that the three lakes at intermediate elevations (Mómelar, Thingvallavatn, Mývatn) tend to have intralacustrine phenotypic differentiation with higher trait dimensionality than other lakes (Fig. 5).

### Intralacustrine diversification: ecosystem size and dimensionality

We found neither the morphospace used by stickleback within a lake nor the occurrence of intralacustrine diversification to be associated with lake size or depth, which are commonly used to approximate ecosystem size (Gavrilets and Losos 2009). This contrasts with theoretical predictions (Simpson 1953; Schluter 2000; Gavrilets and Vose 2005) and empirical findings in other taxa, where positive correlations were found between ecosystem size and the number of species that evolve during adaptive radiations (e.g., Losos and Schluter 2000; Parent and Crespi 2006; Wagner et al. 2014), including Icelandic arctic charr occupying some of the same lakes we studied here (Woods et al. 2012). However, our findings are consistent with studies on Canadian three‐spine stickleback (Vamosi 2003; Ormond et al. 2011), suggesting that stickleback adaptive radiation is unresponsive to ecosystem size. Nonetheless, we found that the dimensionality of phenotypic differentiation was positively related to lake size. Mómelar, being the smallest of our studied lakes is in this regard exceptional as the dimensionality of phenotypic differentiation in its stickleback is comparable to the two largest lakes. Stickleback in this lake may enjoy particularly low interspecific competition as Mómelar is the only lake, where stickleback live in the absence of any other fish species (Lucek *personal observation*). As a consequence, they may have experienced ecological release from interspecific competition and/or predation, facilitating the evolution of phenotypic diversity and phenotypic differentiation in this small and shallow lake (Vamosi 2003; Bolnick et al. 2010; Ormond et al. 2011). Alternatively, Mómelar may harbor additional unique niches and hence provide an increased ecological opportunity.

Consistent with prior studies (Kristjánsson et al. 2002a; Ólafsdóttir et al. 2007a; Millet et al. 2013), we find morphs that are significantly associated with different substrates in lakes Mývatn and Frostastaðavatn, suggesting an important role of habitat heterogeneity in diversification of Icelandic stickleback. Thus, in line with theoretical predictions for evolutionary diversification in heterogeneous habitats (Doebeli and Dieckmann 2003; Gavrilets 2003; Leimar et al. 2008; Débarre 2012), spatial and habitat heterogeneity may drive the relationship between lake size, being a proxy for ecosystem size, and the dimensionality of phenotypic differentiation in our studied systems.

Phenotypic differentiation across the marine–freshwater transition as well as the emergence of intralacustrine phenotypic groups are likely both results of phenotypic plasticity acting in concert with adaptation from standing genetic variation as has been found in stickleback populations outside of Iceland (Baumgartner 1995; Wund et al. 2008; Berner et al. 2011; Lucek et al. 2014b). The relative contribution of plasticity and genetic predisposition varies among these systems and the traits being studied. Nonetheless, plasticity can initially promote differentiation, where subsequent divergent selection among phenotypic groups may then lead to the buildup of genetic differentiation and the emergence of prezygotic isolation (West‐Eberhard 2003; Snorrason and Skúlason 2004; Pfennig and McGee 2010).

### Parallelisms of intralacustrine phenotypic diversification

The traits that are associated with sympatric phenotypic diversification in Icelandic stickleback show parallel trends among lakes, where diversification in body depth occurs in seven lakes, and diversification in pelvic girdle‐related traits in six of nine lakes, one exception being Hraunsfjördur, a former marine fjord that became landlocked only as recently as 50 years ago (Fig. 5; Table S2). Differences in body depth are thought to be of adaptive relevance and have been found among ecologically differentiated stickleback populations in many other systems (Reid and Peichel 2010; Lucek et al. 2013; Ravinet et al. 2013b; Voje et al. 2013), where plankton‐feeding fish are generally more streamlined than benthic feeding fish, facilitating both foraging and cruising in open water (Reid and Peichel 2010). Our observed differentiation in body depth and to a lesser extent in head shape and gill raker length may thus reflect adaptation to different trophic resources (Schluter and McPhail 1992; Walker 1997). Indeed, differences in feeding strategies between sympatric Icelandic stickleback morphs collected from different substrates have previously been found (Kristjánsson et al. 2002a) and may thus importantly contribute to the evolution of phenotypically and potentially ecologically distinct sympatric morphs in Icelandic lake stickleback.

Differences in fin size and antipredator defense traits also occur, especially in the two largest lakes (Fig. 5). Such differences could also be adaptive, where fin sizes relate to different sustained swimming capabilities (Reid and Peichel 2010). Differences in antipredator defense traits may similarly reflect adaptation to different predation regimes, where gape‐limited predators such as birds and fish are thought to select for increased spine lengths (Reimchen 1994) whereas grappling predators like insect larvae select for reduced armor (Reimchen 1980, 1994). However, large predatory insect larvae seem to be rare in Iceland and other selective agents may underlie the observed differentiation in spine lengths (Doucette et al. 2004; Lucek et al. 2012a).

In two instances – the marine population and Frostastaðavatn – the identified phenotypic groups represent different age classes. In Frostastaðavatn, both sexes form distinct age‐related phenotypic groups that differ in body shape and to a lesser extent in defense and head morphology. The individuals assigned to each group are significantly associated with substrates with individuals from the lava substrate being smaller and younger, indicating that habitat choice may differ among age classes. This can itself be adaptive (Dill 1983), where the observed phenotypic differentiation could facilitate habitat and resource partitioning among age classes. Alternatively, two types of stickleback may exist that differ in longevity as has been found in other systems (Baker et al. 2005; Lucek et al. 2012b; Moser et al. 2012).

### Genetic differentiation and reproductive isolation

Neutral genetic population structure within lakes was weak (Table 2), where structure found evidence for genetic structure only in Mývatn (Fig. S2). However, the power of structure to detect genetic clusters from a limited number of markers is constrained when genetic differentiation is weak (Latch et al. 2006; Hubisz et al. 2009). The existence of groups of nonrandomly mating individuals (Wahlund effect), is indicated in four lakes that show significant global inbreeding coefficients (Bernatchez and Wilson 1998). Lastly, pairwise *F*_ST_ suggest significant genetic differentiation between distinct sampling sites in Hraunsfjördur, Thingvallavatn, and Mývatn (Table S1). In the latter case, individuals from Mývatn 1 differ genetically from all other sites, which is congruent with the structure analysis (Fig. S2). In contrast, we found significant genetic differentiation between phenotypic groups only in Thingvallavatn (Table 2). The overall evidence for potential genetic structure among phenotypic groups suggests a further stage of diversification in these lakes (Feder et al. 2012), but contrasts with other studies that found a higher genetic differentiation between populations inhabiting distinct substrates in Hraunsfjördur (Ólafsdóttir et al. 2007b), Mývatn (Ólafsdóttir et al. 2007c; Millet et al. 2013), and Thingvallavatn (Ólafsdóttir and Snorrason 2009). The use of many phenotype‐linked markers in these studies, but few in ours, could account for the differences in the extent of genetic divergence between morphs. Genetic differentiation mainly in phenotype‐linked markers would be consistent with a very early stage along the ecological speciation continuum (Hendry 2009; Feder et al. 2012; Nosil 2012), where divergent selection acts on small regions in the genome but reproductive isolation has not evolved or is too recent to be picked up in allele frequencies at neutral markers (Thibert‐Plante and Hendry 2010).

## Conclusions

Studying Icelandic stickleback, we find that colonization of lakes from the sea is generally associated with an increase in intrapopulation phenotypic variation, which we consider evidence for ecology‐driven diversification. Next, we find that sympatric phenotypic differentiation of morphs within lakes is a recurrent phenomenon among Icelandic lake stickleback, and it involves repeatedly the same phenotypic axes. We suggest this marks a first stage in stickleback lacustrine adaptive radiation, where lake size seems to predict the dimensionality of sympatric phenotypic differentiation among morphs. Finally, we find evidence of neutral genetic differentiation in the two largest lakes. We suggest that this signals a further degree of differentiation, where gene flow between divergent groups is sufficiently constrained, and has been for sufficiently long time to detect differentiation using neutral markers. This is the mark of ecological speciation. Taken together, our data suggest that ecosystem size – approximated by lake surface – predicts the extent of sympatric differentiation and may indicate how far speciation may ultimately proceed in lacustrine stickleback.

## Conflict of Interest

None declared.

## Supporting information


**Figure S1.** Dendrograms based on pairwise Mahalanobis distances among identified phenotypic groups for males and females in Lakes Frostastaðavatn (A), Mývatn (B), and Thingvallavatn (C).

**Figure S2.** Structure analysis for Mývatn combining all sites and sexes.

**Table S1.** Pairwise genetic differentiation (*F*_ST_) between stickleback from all lakes and the marine population (lower triangle) with the respective *P* values based on 1000 bootstrap replicates.
**Table S2.** Pairwise genetic differentiation (*F*_ST_) among sympatric lake sites (lower triangle) with the respective *P* values based on 1000 bootstrap replicates. Significant (*P *<* *0.05) *F*_ST_ values are highlighted in bold.
**Table S3.** Number of identified phenotypic modes and MANOVA results for phenotypic traits using modes as factors, calculated for each lake and for each sex separately.
