# Supplementary figures and images for: Ecosystem size matters: the dimensionality of intralacustrine diversification in Icelandic stickleback is predicted by lake size
Source: Ecol Evol. 2016 Jun 29;6(15):5256–72. doi: 10.1002/ece3.2239 (PMC4984502; doi:10.1002/ece3.2239)

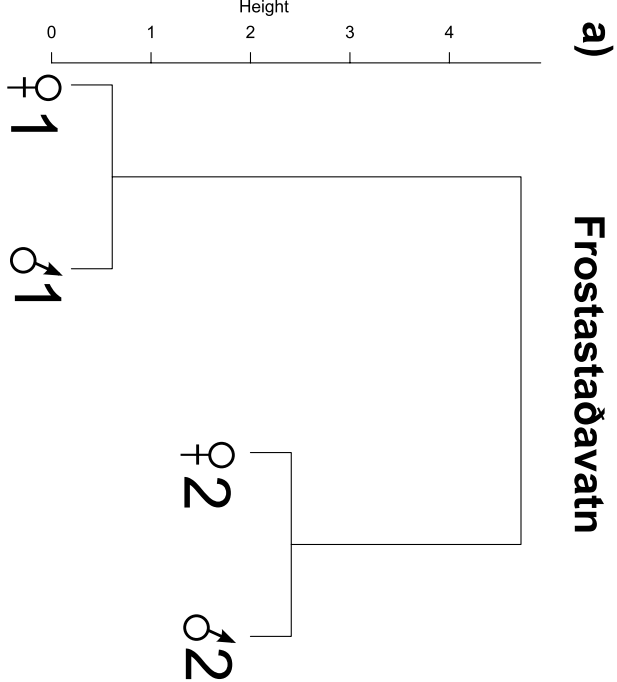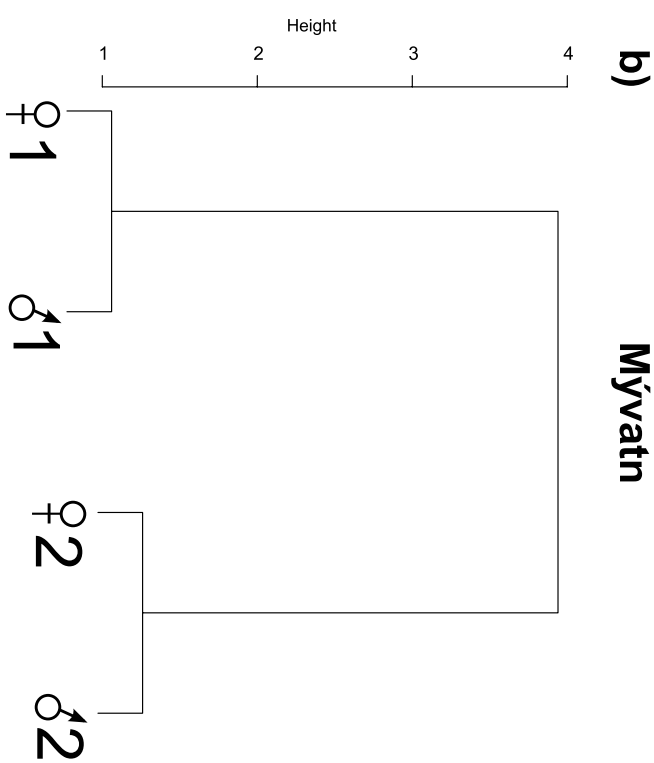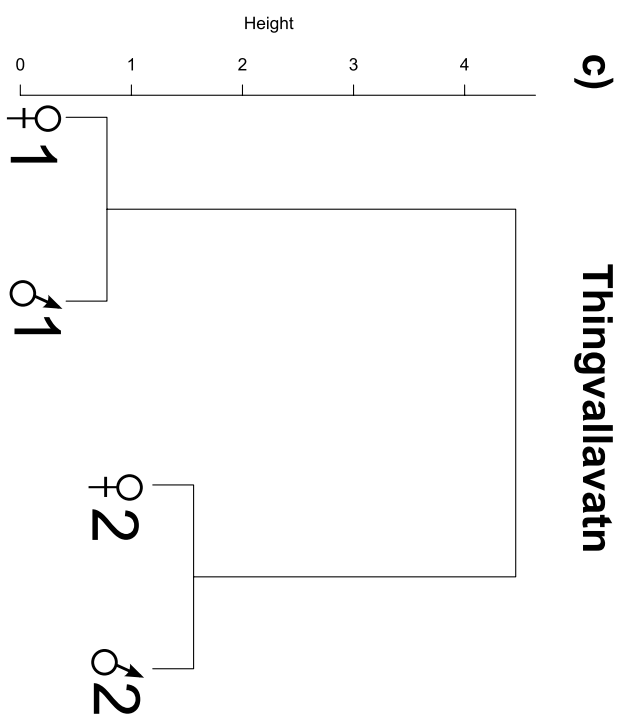

Supplement: Supplementary file 1 — Figure S1. Dendrograms based on pairwise Mahalanobis distances among identified phenotypic groups for males and females in Lakes Frostastaðavatn (A), Mývatn (B), and Thingvallavatn (C). [file ECE3-6-5256-s001.pdf]
