# Supplementary material for: Ecosystem size matters: the dimensionality of intralacustrine diversification in Icelandic stickleback is predicted by lake size
Source: Ecol Evol. 2016 Jun 29;6(15):5256–72. doi: 10.1002/ece3.2239 (PMC4984502; doi:10.1002/ece3.2239)
Supplement: Supplementary file 3 — Table S1. Pairwise genetic differentiation (F ST) between stickleback from all lakes and the marine population (lower triangle) with the respective P values based on 1000 bootstrap replicates. Table S2. Pairwise genetic differentiation (F ST) among sympatric lake sites (lower triangle) with the respective P values based on 1000 bootstrap replicates. Significant (P < 0.05) F ST values are highlighted in bold. Table S3. Number of identified phenotypic modes and MANOVA results for phenotypic traits using modes as factors, calculated for each lake and for each sex separately. [file ECE3-6-5256-s003.docx]

Table S1: Pairwise genetic differentiation (*F*_ST_) between stickleback from all lakes and the marine population (lower triangle) with the respective *p* values based on 1000 bootstrap replicates.

|  | Marine | Hraunsfjördur | Apavatn | Mómelar | Flódid | Galtaból | Mjóavatn | Frostastaðavatn | Mývatn | Thingvallavatn |
| --- | --- | --- | --- | --- | --- | --- | --- | --- | --- | --- |
| Marine | - | 0.001 | 0.001 | 0.001 | 0.001 | 0.001 | 0.001 | 0.001 | 0.001 | 0.001 |
| Hraunsfjördur | 0.033 | - | 0.001 | 0.001 | 0.001 | 0.001 | 0.001 | 0.001 | 0.001 | 0.001 |
| Apavatn | 0.025 | 0.026 | - | 0.001 | 0.001 | 0.001 | 0.001 | 0.001 | 0.001 | 0.001 |
| Mómelar | 0.159 | 0.170 | 0.188 | - | 0.001 | 0.001 | 0.001 | 0.001 | 0.001 | 0.001 |
| Flódid | 0.022 | 0.039 | 0.033 | 0.190 | - | 0.001 | 0.001 | 0.001 | 0.001 | 0.001 |
| Galtaból | 0.360 | 0.353 | 0.389 | 0.547 | 0.322 | - | 0.001 | 0.001 | 0.001 | 0.001 |
| Mjóavatn | 0.300 | 0.275 | 0.310 | 0.462 | 0.243 | 0.143 | - | 0.001 | 0.001 | 0.001 |
| Frostastaðavatn | 0.377 | 0.350 | 0.380 | 0.563 | 0.347 | 0.539 | 0.529 | - | 0.001 | 0.001 |
| Mývatn | 0.306 | 0.296 | 0.283 | 0.472 | 0.303 | 0.576 | 0.554 | 0.511 | - | 0.001 |
| Thingvallavatn | 0.189 | 0.172 | 0.190 | 0.328 | 0.228 | 0.442 | 0.392 | 0.514 | 0.439 | - |

Table S2: Pairwise genetic differentiation (*F*_ST_) among sympatric lake sites (lower triangle) with the respective *p* values based on 1000 bootstrap replicates. Significant (*p* < 0.05) *F*_ST_ values are highlighted in bold.

|  | Hraunsfördur 1 | Hraunsfjördur 2 | Frostastaðavatn 1 | Frostastaðavatn 2 | Mývatn 1 | Mývatn 2 | Mývatn 3 | Mývatn 4 | Mývatn 5 | Mývatn 6 | Thingvallavatn 1 | Thingvallavatn 2 | Thingvallavatn 3 | Thingvallavatn 4 | Thingvallavatn 5 |
| --- | --- | --- | --- | --- | --- | --- | --- | --- | --- | --- | --- | --- | --- | --- | --- |
| Hraunsfördur 1 | - | 0.041 |  |  |  |  |  |  |  |  |  |  |  |  |  |
| Hraunsfjördur 2 | **0.007** | - |  |  |  |  |  |  |  |  |  |  |  |  |  |
| Frostastaðavatn 1 |  |  | - | 0.191 |  |  |  |  |  |  |  |  |  |  |  |
| Frostastaðavatn 2 |  |  | 0.003 | - |  |  |  |  |  |  |  |  |  |  |  |
| Mývatn 1 |  |  |  |  | - | 0.001 | 0.001 | 0.001 | 0.001 | 0.001 |  |  |  |  |  |
| Mývatn 2 |  |  |  |  | **0.066** | - | 0.460 | 0.522 | 0.259 | 0.192 |  |  |  |  |  |
| Mývatn 3 |  |  |  |  | **0.119** | 0.000 | - | 0.594 | 0.342 | 0.430 |  |  |  |  |  |
| Mývatn 4 |  |  |  |  | **0.103** | -0.002 | -0.003 | - | 0.263 | 0.863 |  |  |  |  |  |
| Mývatn 5 |  |  |  |  | **0.104** | 0.004 | 0.002 | 0.003 | - | 0.251 |  |  |  |  |  |
| Mývatn 6 |  |  |  |  | **0.104** | 0.006 | 0.000 | -0.006 | 0.003 | - |  |  |  |  |  |
| Thingvallavatn 1 |  |  |  |  |  |  |  |  |  |  | - | 0.565 | 0.511 | 0.939 | 0.508 |
| Thingvallavatn 2 |  |  |  |  |  |  |  |  |  |  | -0.002 | - | 0.069 | 0.357 | 0.289 |
| Thingvallavatn 3 |  |  |  |  |  |  |  |  |  |  | -0.002 | 0.015 | - | 0.203 | 0.015 |
| Thingvallavatn 4 |  |  |  |  |  |  |  |  |  |  | -0.006 | 0.001 | 0.005 | - | 0.269 |
| Thingvallavatn 5 |  |  |  |  |  |  |  |  |  |  | -0.001 | 0.005 | **0.026** | 0.003 | - |

Table S3: Number of identified phenotypic modes and MANOVA results for phenotypic traits using modes as factors, calculated for each lake and for each sex separately. *P* values for traits are based on ANOVA analyses. Significant *p* values after a Benjamini and Yekutieli correction are highlighted in bold. See main text for abbreviations.

| **Females** |  |  |  |  |  |  |  |  |  |  |
| --- | --- | --- | --- | --- | --- | --- | --- | --- | --- | --- |
|  | **Apavatn** | **Flódid** | **Frostastaðavatn** | **Galtaból** | **Hraunsfjördur** | **Marine** | **Mjóavatn** | **Mómelar** | **Mývatn** | **Thingvallavatn** |
| **Sample size** | 30 | 26 | 70 | 25 | 42 | 24 | 25 | 25 | 95 | 63 |
| **# phenotypic modes** | 2 | 2 | 2 | 2 | 1 | 2 | 1 | 2 | 2 | 2 |
| **MANOVA** | *F*_1,28_ = 15.0 | *F*_1,24_ = 2.5 | *F*_1,68_ = 7.7 | *F*_1,23_ = 1.8 | - | *F*_1,22_ = 12.9 | - | *F*_1,23_ = 22.5 | *F*_1,93_ = 14.3 | *F*_1,61_ = 14.8 |
| ***p*** | **<0.001** | 0.125 | **<0.001** | 0.244 | - | **0.005** | **-** | **<0.001** | **<0.001** | **<0.001** |
| ***Body shape*** |  |  |  |  |  |  |  |  |  |  |
| **Body depth 1** | **<0.001** | **<0.001** | **<0.001** | **<0.001** | - | 0.772 | - | **<0.001** | **<0.001** | **<0.001** |
| **Body depth 2** | **<0.001** | **<0.001** | **<0.001** | **<0.001** | - | 0.849 | - | **<0.001** | **<0.001** | **<0.001** |
| **Caudal peduncle length** | 0.201 | 0.965 | 0.081 | 0.580 | - | **<0.001** | - | 0.051 | 0.680 | **0.002** |
| **Pelvic girdle width** | **<0.001** | **<0.001** | **<0.001** | 0.006 | - | 0.083 | - | **<0.001** | **<0.001** | **<0.001** |
| **Pelvic girdle length** | **<0.001** | **0.001** | **<0.001** | 0.005 | - | 0.022 | - | **0.004** | **<0.001** | **<0.001** |
|  |  |  |  |  |  |  |  |  |  |  |
| ***Spines*** |  |  |  |  |  |  |  |  |  |  |
| **Length 1^st^ dorsal spine** | 0.874 | 0.744 | 0.189 | 0.114 | - | 0.021 | - | 0.014 | **<0.001** | 0.237 |
| **Length 2^nd^ dorsal spine** | 0.518 | 0.911 | 0.012 | 0.081 | - | 0.010 | - | **0.003** | **<0.001** | 0.015 |
| **Pelvic spine length** | 0.727 | 0.578 | 0.691 | **<0.001** | - | 0.236 | - | **<0.001** | **0.007** | **0.001** |
|  |  |  |  |  |  |  |  |  |  |  |
| ***Head shape & trophic morphology*** |  |  |  |  |  |  |  |  |  |  |
| **Snout length** | **0.002** | 0.531 | 0.406 | 0.332 | - | 0.320 | - | **0.005** | **0.002** | 0.011 |
| **Upper jaw length** | 0.186 | 0.078 | 0.825 | 0.244 | - | 0.027 | - | 0.053 | 0.091 | 0.106 |
| **Snout width** | 0.246 | 0.296 | 0.279 | 0.911 | - | 0.533 | - | 0.400 | 0.063 | 0.199 |
| **Length 2^nd^ gill raker** | 0.939 | 0.328 | 0.129 | 0.261 | - | 0.012 | - | **0.002** | 0.084 | 0.371 |
| **Gill arch length** | 0.394 | 0.817 | 0.419 | 0.392 | - | 0.286 | - | 0.280 | 0.185 | 0.356 |
| **Head length** | 0.015 | 0.144 | 0.013 | 0.026 | - | 0.601 | - | 0.080 | **<0.001** | **<0.001** |
| **Eye diameter** | 0.791 | 0.410 | 0.591 | 0.042 | - | 0.267 | - | 0.013 | 0.711 | 0.224 |
|  |  |  |  |  |  |  |  |  |  |  |
| ***Fins*** |  |  |  |  |  |  |  |  |  |  |
| **Total length pelvic fin** | 0.185 | 0.746 | 0.473 | 0.680 | - | 0.129 | - | **<0.001** | 0.042 | 0.034 |
| **Basal length anal fin** | **0.013** | 0.303 | 0.980 | 0.009 | - | 0.023 | - | 0.215 | **<0.001** | **0.004** |
| **Basal length dorsal fin** | 0.200 | 0.747 | 0.079 | 0.104 | - | **<0.001** | - | 0.514 | 0.045 | 0.022 |
|  |  |  |  |  |  |  |  |  |  |  |
|  |  |  |  |  |  |  |  |  |  |  |
| **Males** |  |  |  |  |  |  |  |  |  |  |
|  | **Apavatn** | **Flódid** | **Frostastaðavatn** | **Galtaból** | **Hraunsfjördur** | **Marine** | **Mjóavatn** | **Mómelar** | **Mývatn** | **Thingvallavatn** |
| **Sample size** | 11 | 4 | 58 | 26 | 30 | 14 | 25 | 9 | 90 | 91 |
| **# Modes** | - | - | 2 | 1 | 1 | - | 2 | - | 2 | 2 |
| **MANOVA** | - | - | *F*_1,60_ = 10.6 | - | - | - | *F*_1,23_ = 6.4 | - | *F*_1,88_ = 11.5 | *F*_1,89_ = 15.9 |
| ***p*** | - | - | **<0.001** | - | - | - | **0.003** | - | **<0.001** | **<0.001** |
| ***Body shape*** |  |  |  |  |  |  |  |  |  |  |
| **Body depth 1** | - | - | **<0.001** | - | - | - | 0.299 | - | **<0.001** | **<0.001** |
| **Body depth 2** | - | - | **<0.001** | - | - | - | 0.152 | - | **<0.001** | **<0.001** |
| **Caudal peduncle depth** | - | - | 0.403 | - | - | - | 0.028 | - | 0.916 | 0.346 |
| **Pelvic girdle width** | - | - | **<0.001** | - | - | - | 0.953 | - | **<0.001** | **<0.001** |
| **Pelvic girdle length** | - | - | **<0.001** | - | - | - | 0.118 | - | **<0.001** | **<0.001** |
|  |  |  |  |  |  |  |  |  |  |  |
| ***Spines*** |  |  |  |  |  |  |  |  |  |  |
| **Length 1^st^ dorsal spine** | - | - | 0.012 | - | - | - | 0.104 | - | 0.181 | **0.008** |
| **Length 2^nd^ dorsal spine** | - | - | 0.169 | - | - | - | 0.054 | - | 0.478 | 0.018 |
| **Pelvic spine length** | - | - | 0.222 | - | - | - | 0.058 | - | 0.013 | **<0.001** |
|  |  |  |  |  |  |  |  |  |  |  |
| ***Head shape & trophic morphology*** |  |  |  |  |  |  |  |  |  |  |
| **Snout length** | - | - | 0.533 | - | - | - | 0.643 | - | **<0.001** | **0.004** |
| **Upper jaw length** | - | - | 0.763 | - | - | - | 0.080 | - | 0.284 | 0.031 |
| **Snout width** | - | - | 0.499 | - | - | - | 0.371 | - | 0.587 | **<0.001** |
| **Length 2^nd^ gill raker** | - | - | **0.001** | - | - | - | 0.277 | - | 0.178 | **0.010** |
| **Gill arch length** | - | - | **0.001** | - | - | - | 0.424 | - | **<0.001** | **<0.001** |
| **Head length** | - | - | **0.004** | - | - | - | 0.649 | - | **<0.001** | **<0.001** |
| **Eye diameter** | - | - | 0.516 | - | - | - | 0.552 | - | 0.228 | 0.013 |
|  |  |  |  |  |  |  |  |  |  |  |
| ***Fins*** |  |  |  |  |  |  |  |  |  |  |
| **Total length pelvic fin** | - | - | 0.080 | - | - | - | **0.002** | - | **<0.001** | **0.007** |
| **Basal length anal fin** | - | - | 0.007 | - | - | - | 0.968 | - | **<0.001** | **<0.001** |
| **Basal length dorsal fin** | - | - | 0.085 | - | - | - | **<0.001** | - | **<0.001** | 0.127 |
